# Reduced Genetic Diversity and Increased Structure in American Mink on the Swedish Coast following Invasive Species Control

**DOI:** 10.1371/journal.pone.0157972

**Published:** 2016-06-22

**Authors:** Andrzej Zalewski, Hanna Zalewska, Sven-Gunnar Lunneryd, Carl André, Grzegorz Mikusiński

**Affiliations:** 1 Mammal Research Institute, Polish Academy of Sciences, Białowieża, Poland; 2 Department of Aquatic Resources, Swedish University of Agricultural Sciences, Lysekil, Sweden; 3 Department of Marine Sciences–Tjärnö, University of Gothenburg, Strömstad, Sweden; 4 Grimsö Wildlife Research Station, Department of Ecology, Swedish University of Agricultural Sciences, Riddarhyttan, Sweden; Instituto de Higiene e Medicina Tropical, PORTUGAL

## Abstract

Eradication and population reductions are often used to mitigate the negative impacts of non-native invasive species on native biodiversity. However, monitoring the effectiveness of non-native species control programmes is necessary to evaluate the efficacy of these measures. Genetic monitoring could provide valuable insights into temporal changes in demographic, ecological, and evolutionary processes in invasive populations being subject to control programmes. Such programmes should cause a decrease in effective population size and/or in genetic diversity of the targeted non-native species and an increase in population genetic structuring over time. We used microsatellite DNA data from American mink (*Neovison vison*) to determine whether the removal of this predator on the Koster Islands archipelago and the nearby Swedish mainland affected genetic variation over six consecutive years of mink culling by trappers as part of a population control programme. We found that on Koster Islands allelic richness decreased (from on average 4.53 to 3.55), genetic structuring increased, and effective population size did not change. In contrast, the mink population from the Swedish coast showed no changes in genetic diversity or structure, suggesting the stability of this population over 6 years of culling. Effective population size did not change over time but was higher on the coast than on the islands across all years. Migration rates from the islands to the coast were almost two times higher than from the coast to the islands. Most migrants leaving the coast were localised on the southern edge of the archipelago, as expected from the direction of the sea current between the two sites. Genetic monitoring provided valuable information on temporal changes in the population of American mink suggesting that this approach can be used to evaluate and improve control programmes of invasive vertebrates.

## Introduction

Harvesting of animals in the wild, especially when intense, may lead to the direct extinction of a single population or even a whole species [1]. Besides a drastic reduction in census population size, overexploitation also poses less obvious effects, like changes in effective population size *N*e, due to the increased rate of genetic drift and/or changes in gene flow among demes, or due to a decrease in fitness by selectively removing individuals with specific phenotypic traits from a population [2]. These processes often cause a loss of genetic variation, expressed as a decrease in allelic richness and heterozygosity [3]. Furthermore, harvesting and subsequent reduction of the population size might decrease gene flow between different areas, which affects population structuring, and may further decrease genetic variation. However, a local reduction in population size may sometimes increase the relative number of immigrants into subpopulations, which will increase genetic diversity, and in some cases cause genetic outbreeding and loss of local adaptations [2, 4, 5]. All these effects often reduce recovery rates and increase the extinction risk of the harvested population; these effects have been widely observed both in marine and terrestrial harvested species [2, 6]. A major challenge for managers and conservationists is therefore to establish sustainable harvesting schemes of wild animals that do not negatively affect the demographic and genetic features of the targeted populations [7].

In the case of invasive species, negative genetic and demographic changes in populations may be desired and may provide information that will help to reduce population sizes or even to eradicate particular populations from the wild. As the number of introduced non-native species increases [8] there is a growing need for active management or eradication of specific populations from areas where these developments pose a threat and/or cause high economic damages [9, 10]. Notably, in the literature there are many examples of native populations that have gone extinct (or almost extinct) due to overexploitation [11–13], and many fewer examples of the successful eradication of non-native species [9, 14, 15]. This is partly related to the scale and economic factors of both actions; exploitation of native species usually occurs on a large scale and brings economic profits; eradication of non-native species is usually carried out on a small, local scale and needs large financial support [16, 17]. Despite the fact that both actions have opposite goals (preserve harvested species *vs* eradication of invasive non-native species) they relate to the same ecological theory, suggesting that overexploitation first poses genetic changes that often reduce recovery rates and further increases the extinction risk of the population [18].

As a tool for assessing the outcomes of the management of harvested species, Schwartz *et al*. [7] proposed the use of genetic markers to monitor changes of population genetic parameters. The genetic monitoring approach could provide insights into the temporal changes of demographical, ecological and evolutionary processes in a population in relation to harvesting. Information about most of these processes is difficult, if not impossible, to obtain using traditional methods such as capture-mark-recapture techniques. In contrast to most other genetic studies, which analyse snapshots of population status, genetic monitoring must consider the temporal dimension [7, 19]. Using a temporal scale, genetic monitoring may show the influence of human harvesting on a population in consecutive periods, and therefore may help to plan an effective harvest of the population (an increase or decrease in harvesting rate in relation to temporal genetic changes). The expected results of harvesting should include, a loss of allelic diversity, which in the long term should decrease the ability of a population to evolve and expand, and secondly, a decrease in heterozygosity of invasive species, which in the short term should lead to reducing individual fitness [20]. To monitor the effectiveness of invasive species control, temporal monitoring of changes in genetic parameters should be carried out [21].

Effective management programmes geared toward reducing the population size of an invasive species must also focus on defining management units [21–23]. To understand re-colonisation scenarios, information concerning dispersal and gene flow of the species targeted for control is required. Analysis of gene flow and genetic structure gives the opportunity to both define the management units and the rate of migration between units, as well as to define changes in population structure [21, 24]. Long-term and effective harvesting of non-native species should typically cause a decrease in effective population size, migration rate and an increase in genetic structure in consecutive periods [7]. These parameters (genetic diversity and structuring) may show that invasive species control programmes affect the population and bring expected results; however, during eradication programmes, usually only population size is monitored, to assess the effects of harvesting, with no temporal genetic monitoring [17, 25].

The American mink (*Neovison vison*) is an invasive species that has an impact on native biodiversity and ecosystem functioning, by affecting the populations of both their prey, and their competitors [26–28]. The American mink is endemic to North America and has been introduced into Europe, Asia and South America. In central Europe, American mink was introduced for commercial fur farming, which started to develop in the 1920s and feral populations were established by individuals that escaped from farms [29]. The escaped individuals and their descendants gradually colonized large parts of western and central Europe [29]. Eastern and northern Europe were in turn colonized by descendants of individuals introduced into the wild in Russia in the 1930s. So far, the American mink has colonized up to 28 European countries [29]. The strong negative impact of American mink on native species like water vole (*Arvicola terrestris*), coot (*Fulica atra*) and grebes (*Podiceps* spp.) has been observed on the mainland, but the impact is especially severe on sea islands where American mink reduces the number of seabirds in breeding colonies [26, 28, 30]. Attempts to eradicate mink have been shown to be particularly challenging because of the high ecological plasticity of the species: high reproduction rate, mobility and still ongoing propagule pressure in many areas in Europe [31–33].

To optimize the cost-effectiveness of eradication attempts of American mink based on adaptive management, the development of an effective strategy firstly requires the monitoring of the effects of harvesting on the population, to adapt management action in consecutive periods. In the case of mink, there are essentially two possible temporal trends of an actively harvested population. First, if the genetic parameters of a mink population remain unchanged, this means that the number of removed individuals per season is too low: the population has the capacity to compensate for additional mortality and will therefore indicate no decrease in census population size. Second, if genetic diversity is decreasing, this indicates that the number of removed individuals affects the demography of the mink population, and therefore the population size is expected to decrease. If a mink population is isolated and gene flow is restricted, we may therefore expect the invasive species control to have the desired effect [7].

In this paper we analysed the temporal and spatial variation of microsatellite DNA diversity of American mink inhabiting the west coast of Sweden, to determine the structure and temporal changes of genetic diversity in relation to harvesting. Our goal was to (1) assess the impact of harvesting on allelic richness, heterozygosity and effective population size in consecutive years; (2) analyse the genetic structure of American mink inhabiting the mainland and islands to assess management units; (3) evaluate gene flow from the mainland to the islands to estimate the number of individuals re-colonising the islands.

## Materials and Methods

### Ethical Statement

All procedures involving animals in this study fulfilled the ethical requirement stated by the European directive and Swedish legislation about animals in research. DNA samples were acquired post mortem from invasive American mink killed by licensed hunters (granted by Swedish Environmental Protection Agency) to prevent predation on ground nesting birds in protected areas. Animals were shot during the regular hunting season under the rules of the Swedish hunting law and the DNA-samples were given to us courtesy of the local hunters. Thus, no animals were killed specifically for this study.

### Study area

**Fig 1:**
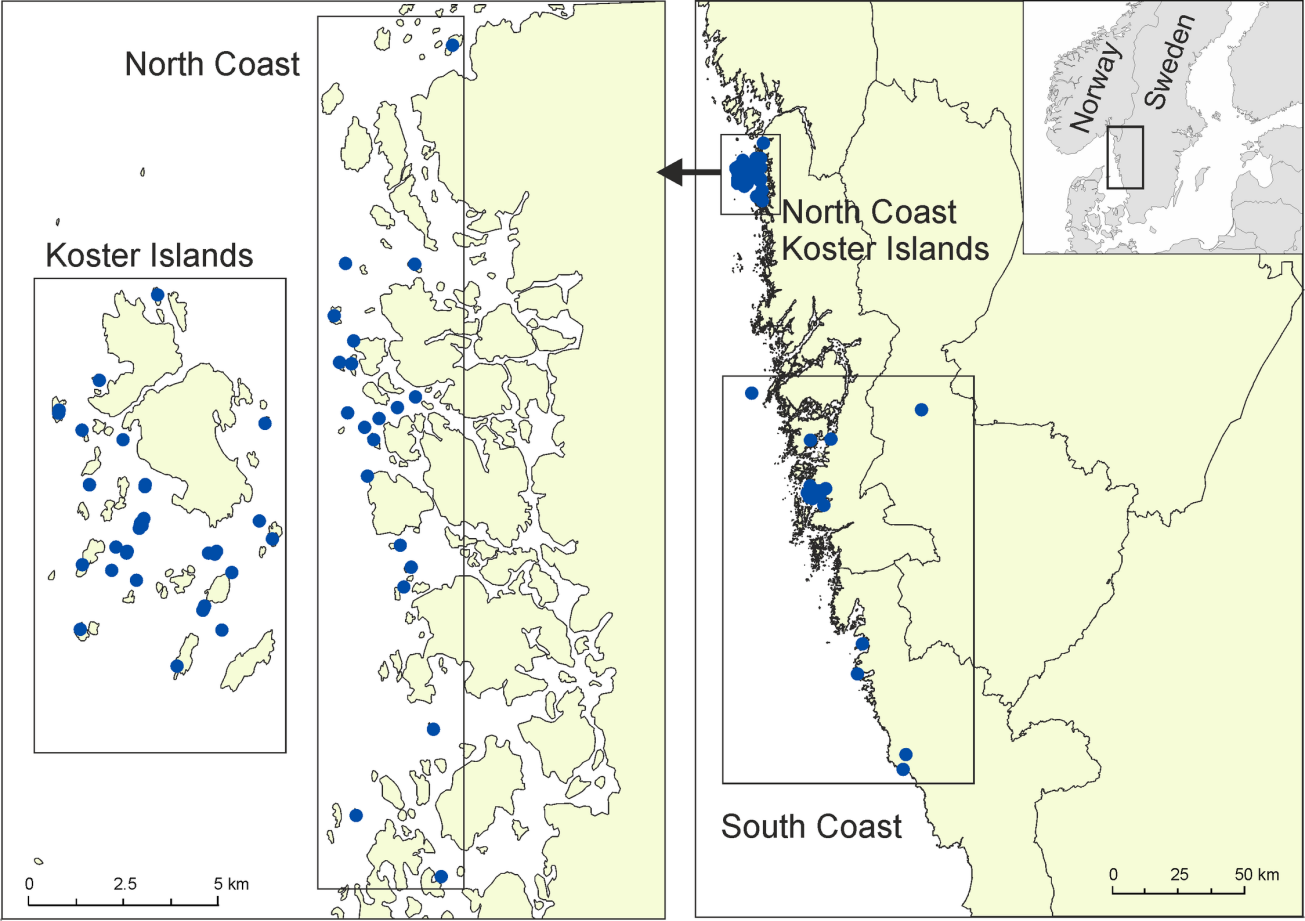
Maps showing the distribution of American mink samples in three study sites (Koster Islands, North Coast and South Coast) in Sweden. As in some locations more than one mink was culled, each point represents the location of at least one culled mink. Background map: Europe Base Map—Level 1 Provinces, AND Products B.V. and AND Data Ireland Limited, ESRI.

To analyse the importance of culling on genetic diversity and gene flow in the American mink, we selected three sites in eastern Skagerrak: (1) a group of islands located 3–4 km from the coast with the shortest distance between islands and coast being 1.6 km (Koster Islands–KI) (N 58°40’; E 11°00’), (2) a mainland site (North coast–NC) located ca. 3 km from the Koster Islands, and (3) another mainland site (South coast–SC) located 30–100 km south of the islands (Fig 1). The sampled areas encompassed approximately 250 km of coastline. Since 2009, Koster Islands have been protected as a national park to conserve very rich and diverse marine ecosystems, habitats and species. The national park area including water is 400 km^2^ and consists of 800 small islands and islets with a total area of 8 km^2^. Most of the park is situated west of a deep water trench. The area also includes two main islands not included in the national park, which have a total area of about 12 km^2^ and are populated by some 300 persons. Common Eider (*Somateria mollissima*), Arctic Skua (*Stercorarius parasiticus*), Arctic Tern (*Sterna paradisaea*), Black Guillemot (*Cepphus grille*), Eurasian Oystercatcher (*Haematopus ostralegus*) and several species of gulls (*Larus spp*.) are examples of marine birds breeding in the park. The presence of American mink is recognised as a major problem for seabird nesting, and the management plan includes an annual hunt of the species to reduce its number.

### Genetic analysis

A total of 205 American mink tissue samples were collected from the islands and coastal areas between 2006 and 2011 (Fig 1). The majority of samples originated from the KI archipelago (132 mink). In addition, we analysed individuals originating from the mainland (46 from NC and 27 from SC). The locations of capture were accurate to 1 km. Mink carcasses were kept frozen until necropsy when a muscle biopsy was taken and placed in concentrated alcohol, and stored at -20°C prior to DNA extraction. We extracted DNA from tissue samples using a DNeasy Blood and Tissue Kit (Qiagen) according to the manufacturer’s instructions. Twenty one microsatellite loci developed for mink were used to genotype individuals: Mvis002, Mvis027,Mvis072, Mvis075, Mvis099, Mvis192, Mvi54, Mvi57, Mvi111, Mvi114, Mvi219, Mvi232, Mvi586, Mvi1006, Mvi1016, Mvi1302, Mvi1321, Mvi1341, Mvi2243, Mvi4001, Mvi4058 [34–39]. Microsatellites were amplified in five multiplex reactions prepared using a Multiplex PCR Kit (QIAGEN) following the manufacturer’s instructions. Reaction mixtures contained approximately 1 μl of template DNA in a total volume of 5.0 μl. The thermal cycle, performed in a DNA Engine Dyad Peltier Thermal Cycler (BIO-RAD), consisted of an initial denaturation step at 95°C for 15 min, followed by 30 cycles at 94°C for 30 sec, 60°C for 1 min 30 sec and 72°C for 1 min and then a final extension period of 30 min at 60°C. The amplified fragments were resolved by electrophoresis using an ABI 3130XL Genetic Analyzer (Applied Biosystems) using GeneMarker 1.85.

Evaluations of the presence of null alleles were performed using MicroChecker version 2.2.3 [40]. Loci that consistently departed from Hardy-Weinberg equilibrium, showed linkage equilibrium or evidence of null alleles were removed from further analyses (one locus only–see results). For each pair of loci in each population, estimates of pairwise linkage disequilibrium, departures from the Hardy-Weinberg equilibrium and inbreeding coefficients (*F*_IS_) were calculated using GenePop on the Web version 4.2 [41] and Bonferroni’s correction was applied to multiple comparisons. The genetic variability of each locus within each site was estimated as the mean allele number (*A*), mean number of private alleles (*A*_private_), observed heterozygosity (H_O_) and unbiased expected heterozygosity (uH_E_) using FSTAT 2.9.3 [42] and GenAlex version 6.5 [43]. The mean number of alleles per locus is expected to be sensitive to sample size; therefore, estimates of the expected allele number per locus were corrected for unequal sample size (*Ar*) using FSTAT.

We used a range of different analytical approaches for identifying genetic differentiation across samples of American mink. Population genetic structure was first detected by the determination of *F*_ST_ levels among predefined populations using FSTAT, and the determination of the recently developed alternative measure of genetic differentiation D_est_ [44] using the software SMOGD 1.2.5 [45]. Second, hierarchical population genetic structure was assessed to test for genetic homogeneity, with the analysis of molecular variance (AMOVA) using ARLEQUIN v.3.5.1.2. Levels of significance were based on 10 000 random permutations.

The genetic structure of American mink was further assessed using individual based analyses with the software STRUCTURE 2.2 [46], and a discriminant analysis of principal components (DAPC; [47]). The greatest rate of change of the likelihood function with respect to the number of clusters *K* (ΔK) was used to find the most likely *K* [48]. For each round of STRUCTURE we used the model that assumes no prior information about the location and the admixture model with correlated allele frequency parameters (λ = 1), and a burn-in phase of 50,000 interactions followed by a run phase of 50,000 interactions. Posterior probability values for the number of clusters (*K*), ranging from 1 to 7, were calculated from 10 independent runs to establish consistency. This method usually detects only the uppermost level of genetic structure [48], and in the first round of STRUCTURE we searched for the number of genetically different clusters using the entire data set. To assess hierarchic structure at a lower level we made further runs of STRUCTURE for subsets of individuals assigned to the separate clusters in the previous run. When the rate of change of the likelihood function indicated that the most probable K-value was equal to one, we treated this cluster as the bottom level of structuring.

Finally, cryptic genetic structures were analysed using a discriminant analysis of principal components (DAPC; [47]). DAPC provides a description of the genetic structuring using coefficients of alleles in linear combinations that produce the largest between-group and smallest within-group variances in these loadings. This analysis detects clusters within the genetic data without the assumptions of HW proportions or linkage equilibrium [49]). First, we established the most likely number of genetic clusters associated with the lowest Bayesian Information Criterion (BIC) values, computed with the **find.clusters** function in adegenet 3.1.9, an R package dedicated to the multivariate analysis of genetic markers [49]. In these analyses we covered numbers of clusters between 1–30 following the procedure outlined in Jombart *et al*. [47]. Retaining too many PCs in DAPC can lead to overfitting of the discriminant functions; therefore, we performed DAPC retaining the optimal number of PCs based on the calculation of the α-score, which measures the difference between the proportion of successful reassignment of the analysis (observed discrimination) and values obtained using random groups (random discrimination). The optimization α-score analyses show that only seven PCs needed to be retained for the assignment analysis (S1 Fig). Therefore, next we performed DAPC analyses using the **dapc** function in adegenet retaining a conservative seven PCs.

We assessed the degree of dispersal (number of migrants) between sites using two different methods. First, we estimated the proportion of individuals with membership q≥0.8 in the first level of the structure analysis conducted with STRUCTURE (see above). Second, we estimated first generation migrants by genetic assignment testing of an individual to a population other than where it was actually collected, implemented in GeneClass2 version 2.0; [50]). We conducted the assignment test using Bayesian probability methods and Monte-Carlo resampling for a probability test with 10 000 simulated individuals and a threshold level of 0.03 in GeneClass2.

We also estimated current rates of gene flow between the sites using a Bayesian MCMC method that relaxes the Hardy-Weinberg equilibrium assumption by using population-specific inbreeding coefficients, implemented in BIMR 1.0 [51]. Ten replicates were performed for each MCMC run of 1000 iterations with 10000 samples and a thinning interval of 50 for each of the 10 replicates. Each of the 10 replicates started with 20 short pilot runs of 1000 iterations each in which incremental values were tuned by the program in an effort to obtain acceptance rates between 25% and 45%. We then chose the run with the lowest Bayesian deviance (D_assign_) calculated by BIMR to extract parameter estimates. Density functions were analysed and the mode (point estimate) and 95% highest posterior density interval (HPDI) were noted.

The linkage disequilibrium (LD) and molecular coancestry (MC) approaches (NeEstimator v. 2; [52]) were used to estimate contemporary effective population size (*N*e) for each of the three geographic sites. The samples of mink were pooled into two culling season groups to get reasonable samples sizes. Effective population size is a crucial parameter in the management and eradication of invasive species because of its influence on population viability and the ability to predict extinction risk [19]. The linkage disequilibrium method builds on the expectation, that in an isolated population, the correlation of unlinked alleles at unlinked loci arises from genetic drift [53]. As suggested by Waples and Do [54], alleles that had a frequency below 0.05 were omitted from the analysis, and for all analyses a random mating model was assumed and 95% jackknife confidence intervals were assessed.

## Results

### Number of removed mink

**Fig 2:**
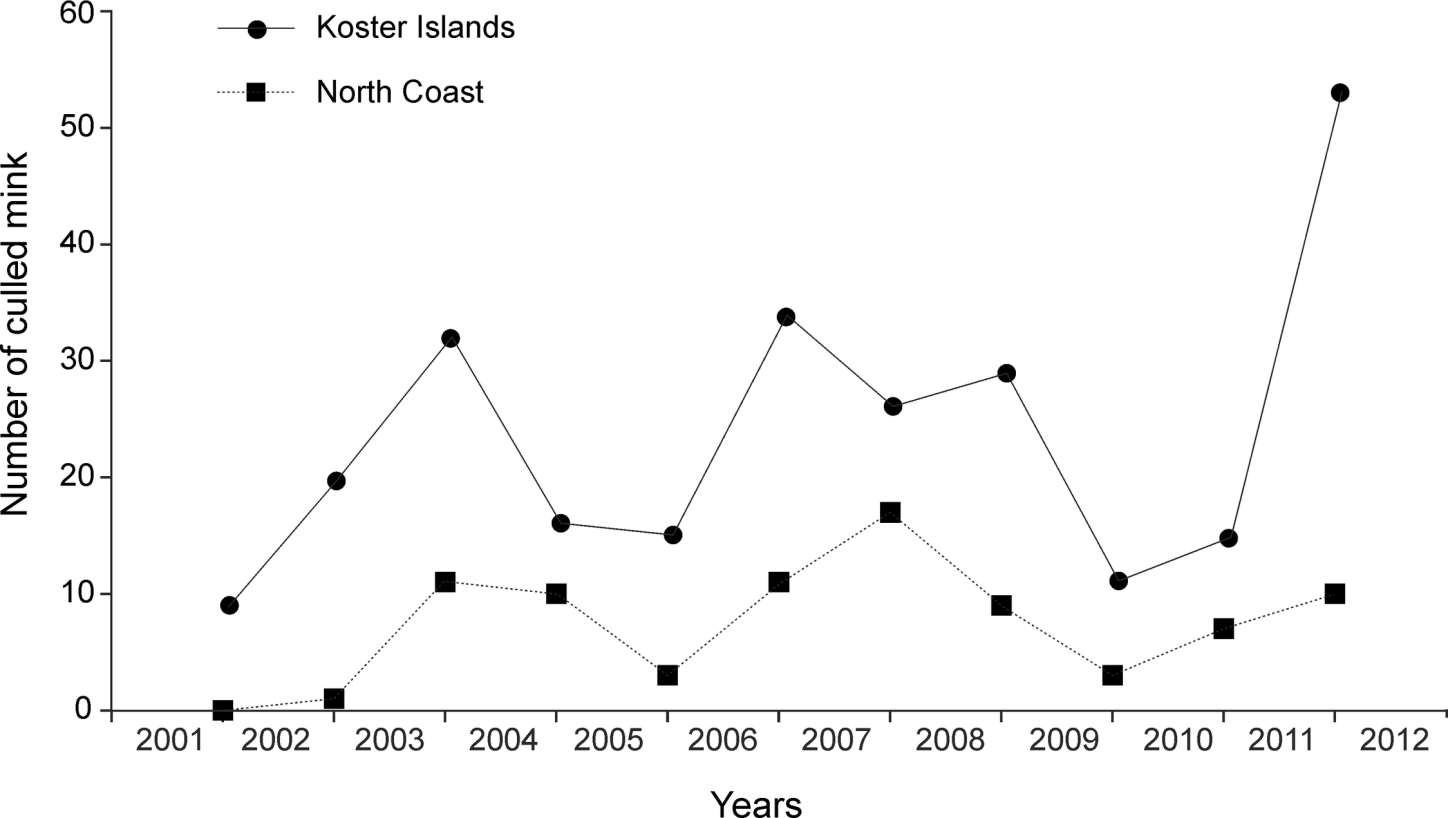
Number of American mink removed from Koster Islands and the North Coast in consecutive culling seasons.

During eleven years (2001–2012) two hunters removed 260 mink from KI and 82 from the NC site. These hunters removed from 9 to 53 (on average 23.6) mink per hunting season on KI and a maximum of 17 (on average 7.5) mink from the NC site (Fig 2). An additional hunter operated only on KI and he removed approximately between 20–40 mink per year. The hunters removed a minimum of 4 mink /km^2^ of island in the KI area per hunting season. Males were culled at a higher proportion than females at all sites: on KI 59.5% of culled mink were males, NC– 57.8% and SC 73.1%.

### Genetic diversity

Twenty one loci were genotyped for 205 individuals with 1.2% missing data in the final data set from three study sites. Significant presence of null alleles (>20% in each site) was found in one locus (Mvi1302), which was subsequently excluded from further analysis. Fourteen of 2090 pairwise locus exact tests of linkage disequilibrium were significant after Bonferroni correction (P < 0.00002), but these were scattered randomly across locus pairs. All 20 microsatellite loci were polymorphic and overall a total of 166 alleles were found, with an overall mean of 8.3 (SE±0.45). The total number of alleles per locus ranged from 5 (Mvis002) to 12 (Mvi114 and Mvis099). The mean number of alleles (*A*) per locus within the sampling sites and years ranged from 4.1 to 6.3, the mean number of private alleles (*A*_private_) from zero to 0.6 and the allelic richness (*Ar*) from 3.3 to 4.3 (Table 1). In KI, both the number of alleles (*A*) and allelic richness (*Ar*) decreased significantly in consecutive years of mink eradication from 6.3 in 2006 to 4.1 in 2010 and from 4.5 to 3.5 respectively (Friedman test, p<0.001; Table 1). The number of alleles and allelic richness varied only slightly among years of eradication at the NC site (Friedman test p = 0.52 and 0.20 respectively), and allelic richness was not significantly different between the North and South Coast sites (Friedman test, p = 0.317). The decrease of allelic diversity in KI was especially marked in rare alleles (with frequency ≤ 0.07), whose number decreased from 53 in 2006 to 25–22 in 2010–2011. In NC rare alleles varied between 26 and 35 in various years.

**Table 1:** Genetic diversity indices of American mink from three sites in Sweden collected in consecutive years 2006–2011. N–number of analysed samples; A–mean number of alleles per locus (direct count); *Ar*–allelic richness estimated by rarefaction based on a minimum sample size n = 7; Rare *A*–number of alleles with frequency ≤ 0,07 across all loci; *A* private–private alleles, H_O_−observed heterozygosity; uH_E_−unbiased expected heterozygosity. Year reflects the hunting season (e.g. 2006 indicates the hunting season of 2006/2007). P value after Bonferroni correction 0.0045.

Site	Code	Year	N	*A*	*Ar*	Rare *A*	*A* private	H_O_	uH_E_	Overall F_IS_	HWE (P-value)
Koster Islands	KI	2006	30	6.30	4.53	53	0.05	0.671	0.689	0.026	0.0004
	KI	2007	24	5.45	4.11	42	0.25	0.640	0.648	0.012	0.0448
	KI	2008	29	5.25	3.94	39	0.05	0.641	0.625	-0.026	0.0001
	KI	2009	11	4.35	3.84	22	0.00	0.664	0.610	-0.093	0.5915
	KI	2010	10	4.05	3.76	15	0.00	0.600	0.614	0.024	0.7789
	KI	2011	28	4.45	3.55	22	0.10	0.589	0.584	-0.008	0.7611
North Coast	NC	2006	10	5.05	4.56	26	0.00	0.604	0.704	0.149	0.0129
	NC	2007	15	5.50	4.47	35	0.15	0.635	0.662	0.041	0.0355
	NC	2008–2009	12	5.00	4.27	28	0.00	0.619	0.654	0.055	0.0098
	NC	2010–2011	9	5.25	4.84	35	0.10	0.749	0.730	-0.027	0.8245
South Coast	SC	2010–2011	27	6.30	4.57	46	0.55	0.631	0.694	0.093	0.0001

All sampling sites showed intermediate values of heterozygosity: H_O_ and uH_E_ per site in various years ranged from 0.589 to 0.749 and from 0.584 to 0.730 respectively (Table 1). In the KI, the H_O_ and uH_E_ decreased in consecutive years of eradication from 0.671 to 0.589 and from 0.689 to 0.584 respectively; however in contrast to H_O_, only uH_E_ decreased significantly (Friedman test, p = 0.234 for H_O_ and p = 0.0003 for uH_E_). At the NC site both indices were similar among years and similar to SC (Friedman test, p>0.05). Three of the 11 temporal sampling sites (KI2006, KI2008 and SC) showed significant deviations from Hardy–Weinberg expectations after Bonferroni correction. In all instances this was due to a deficiency of heterozygote genotypes. Single-locus Hardy–Weinberg equilibrium tests showed that heterozygote deficits were attributable to the locus Mvis586 in the NI2006 sampling sites, locus Mvi114 in the NI2008 sampling sites, and locus Mvi4058 in the SC sampling site.

### Spatial and temporal genetic structure

**Fig 3:**
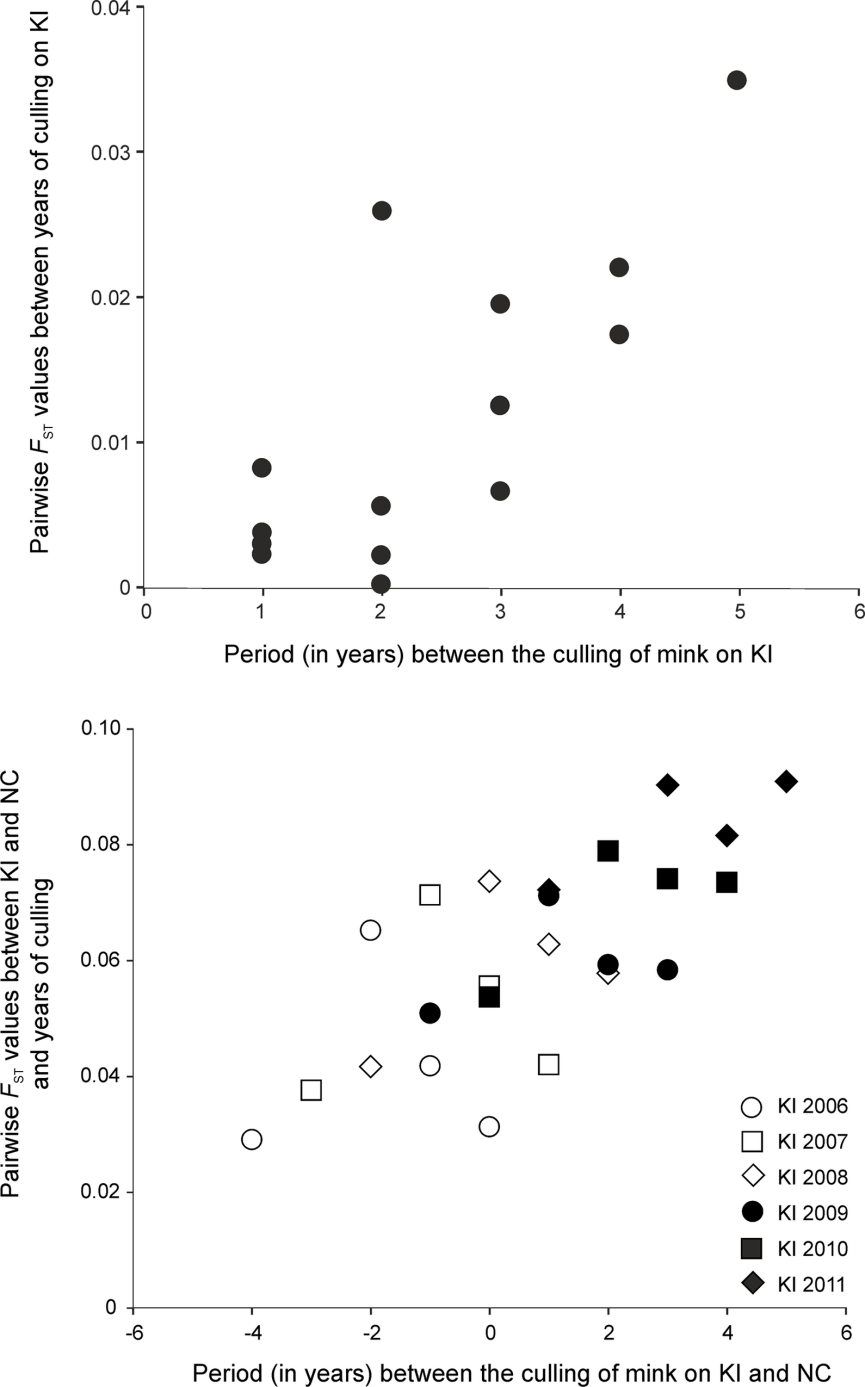
**(upper panel) The relationship between genetic differentiations (pairwise *F***_**ST**_**) between consecutive years and the period (in years) between the culling of mink on the Koster Islands; (lower panel) the relationship between genetic differentiations (*F***_**ST**_**) between consecutive years and the period between the culling of mink on the Koster Islands and North Coast.** Period calculated as year of sampling on Koster Island minus year of sampling on North Coast. Values of *F*_ST_ from S1 Table.

Larger *F*_ST_ values (0.031–0.116) were observed from comparisons between KI and both coast sites than between years within sites (S1 Table). Within each of the two sites (KI *vs* coast) only 3 of 30 comparisons indicated no significant divergence after sequential Bonferroni correction, suggesting that gene flow restriction occurs between the islands and the coast. Differentiation was the highest between KI and SC (0.077–0.116) and moderate but significant differentiation was also observed between NC and SC (0.065–0.092). In the mink population that inhabits KI, the differentiation also increased between consecutive years of culling, from 0.003 between 2006 and 2007, to 0.030 between 2006 and 2011, suggesting genetic drift at this site (Fig 2, S1 Table). The genetic differentiation of mink from KI and NC significantly increased over the time of collection of the mink samples (Fig 3, S1 Table), suggesting that control of mink numbers at both sites affected the structuring of these populations. Similar results were obtained using D_est_ estimator. Between years at the same site, the D_est_ level ranged from 0.001 to 0.041, and between KI and NC varied from 0.02 to 0.12. Differentiation between KI and SC was the highest and ranged from 0.134 to 0.174 (S1 Table).

A hierarchical analysis of molecular variance (AMOVA) was used to assess the distribution of genetic variation at temporal and spatial scales. AMOVA indicated that only 1.5% of overall variance was attributable to differences among years within sites, with 6.8% of variance attributable to differences between the sites (Table 2).

**Table 2:** Results of hierarchical AMOVA comparing the genetic variation of American mink over 6 years within 3 sites in Sweden.

Source of variation	d.f.	Sum of squares	Fixation index	Percentage of variation	P-value
Among sites	2	108.454	Φ_CT_ = 0.068	6.76	<0.001
Among years within sites	8	71.375	Φ_SC_ = 0.016	1.46	<0.001
Within years	399	2282.335	Φ_ST_ = 0.082	91.78	<0.001
Total	409	2462.163			

**Fig 4:**
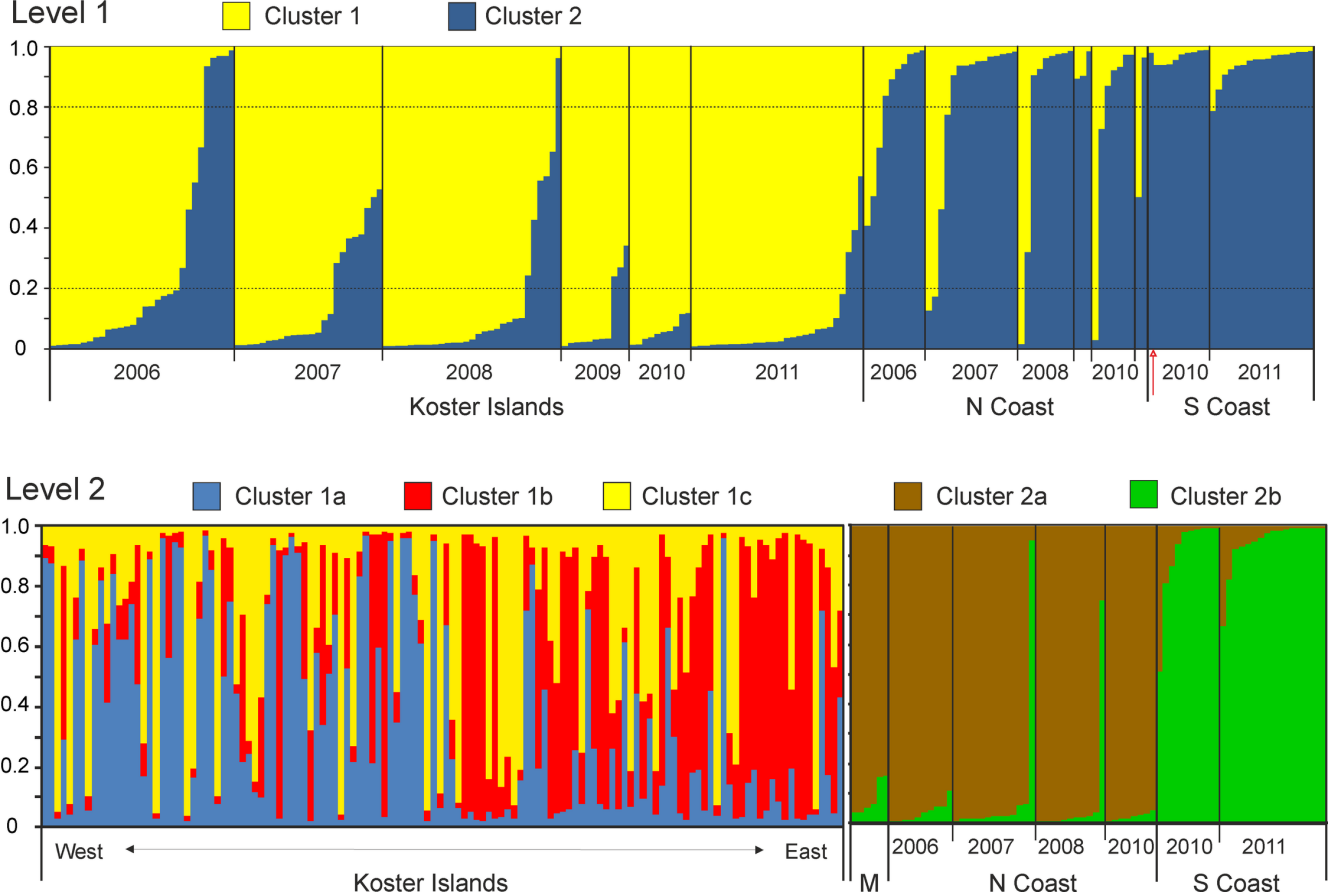
Bayesian assignment of American mink from Sweden in the genetic clusters identified by STRUCTURE analysed in two hierarchical levels. Each American mink is represented by a single vertical bar. The locality of origin and year of capture for each individual is indicated below. Dashed black lines indicate the threshold probabilities of 0.80 and 0.20, used to assign each individual to a single cluster. Red arrow indicates one individual from the South Coast which was caught in 2007 and was included with the group of mink caught in 2010. M–indicates individuals from Koster Island assigned as migrants from the coast at level 1.

The clustering approach used by STRUCTURE to determine the number of genetic groups across the entire data set identified two hierarchical levels of subdivision. Initial partitioning of the data indicated the presence of two clusters (Fig 4; S2 Fig). Most individuals from KI were assigned to one cluster (indicated by yellow) and most individuals from both coast sites were grouped into a second cluster (indicated by blue; Fig 4 and S2 Table). The average assignment of individuals from KI to cluster 2 decreased in consecutive years of eradication from on average 0.281 in 2006 to 0.08 in 2011. A second round of STRUCTURE analysis conducted separately for samples assigned to the two different clusters indicated additional substructures in both clusters. In cluster 1 (KI), three genetic clusters (1a, 1b and 1c) and in cluster 2 (the coast sites) two clusters (2a and 2b) were detected (Fig 4; S2 Fig). No clear partitioning of samples collected from KI was supported by the second round of STRUCTURE, as many individuals in consecutive years were strongly admixed between the three clusters (S2 Table). However, individuals collected further west in KI were often grouped to a different cluster than individuals collected from islands in the east (closer to the coast) (Fig 4, S3 Fig). The average assignments of individuals from the western islands into three clusters (1a, 1b, 1c) were 0.463, 0.205, 0.332 respectively, whereas from the eastern islands they were 0.211, 0.503, 0.285, which indicated some geographical structure among individuals at this site (S3 Fig).

The second round of STRUCTURE analysis for the assignment of individuals to cluster 2 split mink from both coast sites. The individuals from the NC were clearly grouped into one cluster (2a), and separated from individuals from the SC, which were grouped into another cluster (2b) (Fig 4). For individuals from NC, the average proportion of membership q to cluster 2a was 0.933, and for individuals from SC to cluster 2b was 0.929. Migrants from KI were assigned to cluster 2a with an average q of 0.915. Following two rounds of analysis, a total of 5 clusters of American mink were identified, which partially reflected their geographical distribution.

**Fig 5:**
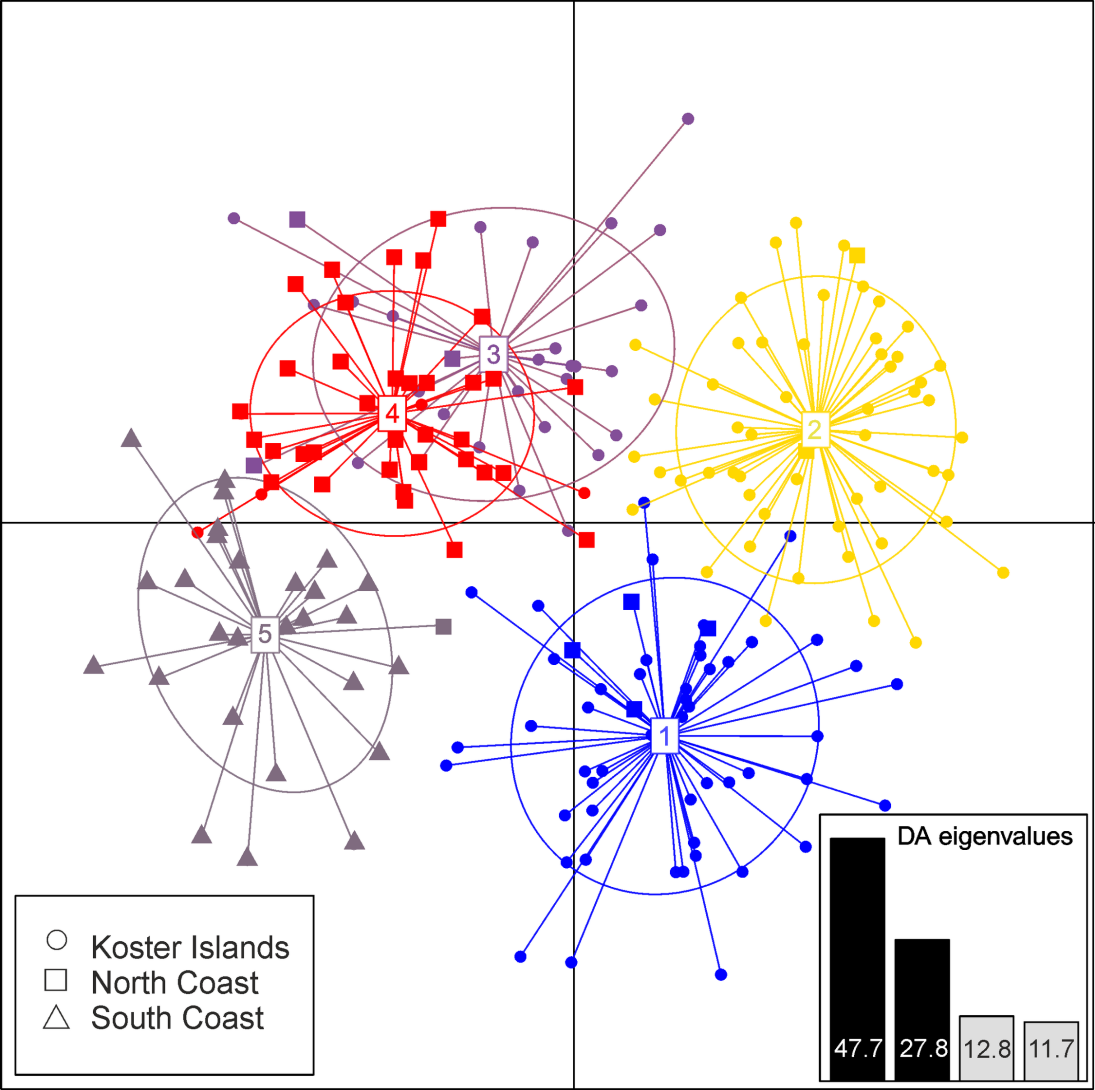
Discriminant analysis of principal components (DAPC) of American mink from Sweden grouped into 5 clusters (various colour) on the first two axes of DAPC. The main graph plots show the first two discriminant axes (explaining 47.7 and 27.8% of the variation respectively). Each point represents an individual, colour indicates the assignment of an individual to a cluster, whereas shape indicates the site of origin of an individual.

Similar to analyses in STRUCTURE, the DAPC clustering algorithm based on BIC suggested five distinct clusters (Fig 5, S4 Fig, S2 Table). The two main axes of the DAPC analysis explained 75.5% of the total variability among clusters (Fig 5). The KI samples were separated from SC and NC by the first axis of the DAPC, and samples from KI were separated into three clusters by the second axis, but these clusters showed no observable relationship with the sampling year. Mink from KI were however assigned to these three clusters in high proportion (93.3–100%; S2 Table). There was no evidence of admixture between SC and the two other sites. The scatterplot of clusters showed considerable overlap between one cluster of samples from KI (cluster 3) and samples from NC (cluster 4), suggesting the presence of potential first generation migrants identified at both sites (Fig 5, S2 Table).

### Number of migrants and effective population size

**Fig 6:**
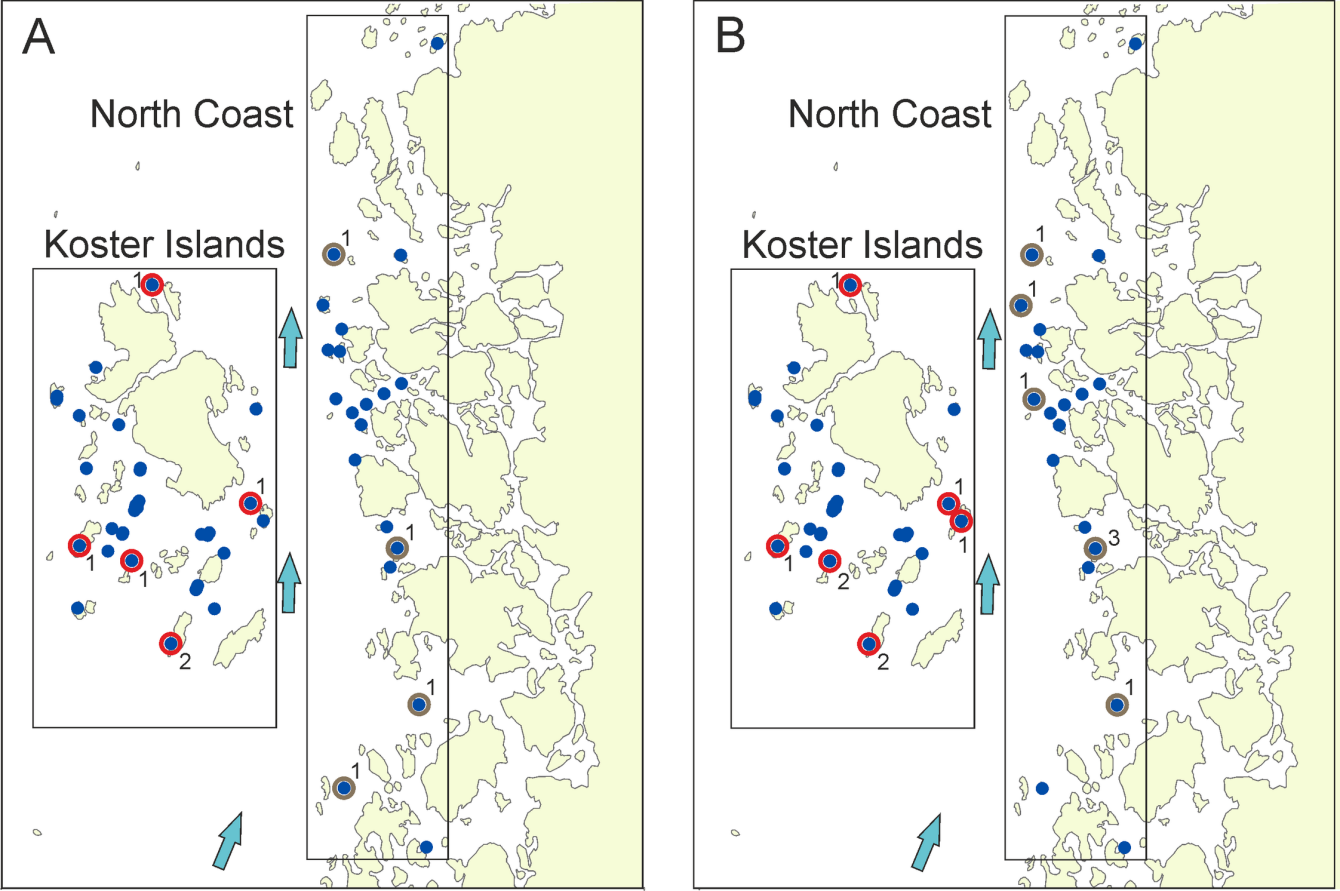
Distribution of all American mink samples and mink marked as migrants from the North Coast to Koster Islands (red circles) and from Koster Islands to the North Coast (brown circles). The migrants were assigned by two methods: STRUCTURE with q ≥ 0.8 (panel A), GeneClass2 with probability ≤ 0.03 (panel B). Numbers indicate the number of migrants at each location. Arrows indicate the direction of sea current. Background map: Europe Base Map—Level 1 Provinces, AND Products B.V. and AND Data Ireland Limited, ESRI.

Two different methods indicated recent migrants between sites. Based on STRUCTURE assignment tests at the first level, 6 mink (4.5%) sampled from KI were assigned to the NC group, whereas 4 mink (8.7%) from NC were assigned to a group of mink from KI, with thresholds q>0.8 (Table 3). At the second level, one (2.2%) individual from NC was assigned to SC, but no individuals were assigned in the opposite direction. Assignment tests computed in GENECLASS2 indicated that in KI 8 (6.1%) individuals were assigned to NC, whereas there were 7 (15.2%) individuals from NC assigned to KI. All these analyses indicated recent movements of individuals between sites, with an asymmetric character; a more than two times higher proportion of migrants from KI was found at NC, than in the opposite direction. Furthermore, in the two methods, 12 out 15 (80%) assigned migrants with known sex were males, suggesting strong male-biased dispersal (Table 3). More migrants from NC were culled on the south part of the KI, and migrants in the opposite direction, from KI, were culled in the northern part of NC (Fig 6).

**Table 3:** The proportion of migrants and the number of males and females dispersed between sites. M–males; F–females. Information about sex was not available for all collected mink samples.

Method	From Koster Islands to N Coast		From N Coast to Koster Islands		From N Coast to S Coast	
	% of migrants	M; F	% of migrants	M; F	% of migrants	M; F
Structure	8.7	3; 1	4.5	4; 1	2.2	1; 0
GenClass	15.2	6; 1	6.1	4; 2	2.2	1; 0

The run with the lowest Bayesian deviance (*D*_assign_) indicated that the mean migration rate between the three sites ranged from almost no migration into SC from the two other sites, to as high as 14.9% from KI to NC (Table 4). We also identified the movement of mink from KI to NC to be asymmetric. Although the 95% HDPIs overlapped for both pairwise estimates, we observed higher migration from KI to NC (an average of 14.9% of the population had emigrated within the last generation) than from NC to KI (average 9.8%). There were asymmetric movements of mink between SC and both other sites; we observed low but higher movement from SC to both other sites than in the opposite direction.

**Table 4:** Recent migration rate (Nm) of American mink between three sites in Sweden. Estimations are based on posterior means and modes calculated by BIMR. Within-site movement is marked in bold.

Into/From		Koster Islands	N Coast	S Coast
Koster Islands	Mean	**0.880**	0.098	0.021
	Mode	**0.887**	0.096	0.014
	(95% HDPI)	**(0.759; 0.943)**	(0.033; 0.219)	(0.001; 0.108)
N Coast	Mean	0.149	**0.825**	0.025
	Mode	0.144	**0.832**	0.013
	(95% HDPI)	(0.031; 0.316)	**(0.647; 0.947)**	(0.006; 0.139)
S Coast	Mean	1.08^−8^	1.08^−8^	**1**
	Mode	1.41^−8^	7.6^−9^	**1**
	(95% HDPI)	(3.39^−10^; 2.09^−08^)	(7.64^−10^; 2.13^−08^)	**(1;1)**

The effective population size was lower in KI than at the two other sites. Over the entire sampling period (2006–2011), the linkage disequilibrium method gave an average effective population size (*N*_e_) of 29.1 in KI (95% confidences interval 23.5–36.0) and 44.0 at NC (95% confidences interval 34.6–58.2) (Table 5). Based on analyses of temporal subsets of data (2 year periods), *N*_e_ ranged from 20.2 to 25.5 in KI and from 17.5 to 70.8 in NC, and no decrease in *N*_e_ between years of mink control (2006–2011) was indicated. The estimation of *N*_e_ based on the molecular coancestry method was from two to five times lower in comparison to the linkage disequilibrium method, but the proportion of *N*_e_ between sites was similar: *N*_e_ was highest at SC, moderate at NC and lowest at KI (Table 5).

**Table 5:** Estimates of effective population size (*N*_e_) derived with different measures for American mink samples collected in the years 2006–2011 in Sweden. LN–Linkage disequilibrium method, MC–Molecular coancestry method.

			Effective population size (95% CI for *N*_e_)	
Site	Year	N	LD	MC
Koster Islands	2006–2007	54	20.2 (16.9–24.2)	5.8 (3.7–8.4)
	2008–2009	40	20.9 (16.1–27.6)	4.9 (3.2–6.9)
	2010–2011	38	25.5 (19.1–35.6)	7.4 (3.7–12.4)
	2006–2011	132	29.1 (23.5–36.0)	5.2 (3.4–7.4)
North Coast	2006–2007	25	28.1 (20.8–40.8)	14.0 (4.6–28.7)
	2008–2009	12	17.5 (11.0–33.1)	3.7 (2.0–5.9)
	2010–2011	9	70.8 (30.6- ∞)	13.4 (4.0–28.4)
	2006–2011	46	44.0 (34.6–58.2)	18.0 (3.7–43.3)
South Coast	2010–2011	27	35.3 (26.1–51.3)	23.4 (4.8–56.5)

## Discussion

In this study we demonstrate that genetic monitoring can be used to evaluate the effectiveness of culling of invasive American mink. We used temporal microsatellite DNA data to test the hypothesis that culling mink from small islands has altered genetic diversity and effective population size. In consecutive years of mink culling on Koster Islands the allelic diversity decreased, genetic structuring in a temporal and spatial scale increased, but the effective population size did not change. In contrast, in both mainland populations, no temporal changes in genetic diversity and structuring were observed, suggesting genetic stability of these populations over six years of culling. Across all years, effective population size was higher at the mainland sites than on the Koster Islands.

The eradication or population control of non-native invasive species has been carried out in many different localities on several continents [9, 10]. In some of these programmes genetic analyses were used, but mainly for the identification of genetically different management units, assessing the numbers or origin of migrants from neighbouring areas, or identifying ongoing propagule pressure [55–58]. Despite the fact that many studies have analysed temporal changes in genetic diversity in over-harvested populations (e.g. fish or ungulates) or endangered species [2, 59–61], genetic monitoring has rarely been used in programmes of non-native species population control. However, as demonstrated in this study, the temporal monitoring of genetic diversity can provide valuable insights into the effectiveness of non-native species control that are difficult to obtain using other methods.

Overall, the genetic diversity of introduced non-native species is related to propagule pressure: mainly it is related to the number of introduction events and the number of introduced individuals [32, 62–64], as well as the genetic composition of the introduced individuals. If introduced individuals originated from several sites of the native range of the species, genetic diversity could be higher than if they originated from a single site [64, 65]. After introduction, the genetic diversity may decrease, especially in isolated populations, due to the gradual decline of population size and increased genetic drift [66]. At the start of the present study in 2006, the allelic richness was at a similar level on KI as at the coast sites, which suggests ongoing gene flow between these sites. This was also confirmed by the observation of recent immigrants from NC to KI (Fig 4). Six years later, we observed a clear decline in genetic diversity on KI, while no change was detected at the coastal sites. In the following years of mink culling allelic richness and heterozygosity decreased (36% and 15% respectively). In contrast, at NC genetic drift is possibly compensated for by gene flow from populations further inland, and culling did not affect genetic diversity, or alternatively effective population size is simply larger and loss of genetic diversity slower. The decrease of genetic diversity on KI was caused by a decreasing number of rare alleles, with frequencies lower than 7% at the end of the study period. A strong decline in allelic richness and loss of rare alleles has been previously observed when population size is reduced [66]. Temporal reduction in genetic diversity (allelic richness and heterozygosity) has been demonstrated in many overharvested populations of mammal and fish species [67–69]. A similar pattern has also been observed for species whose density has largely decreased due to human-induced changes in ecosystems—for example in ocelot (*Leopardus pardalis*) or red squirrel (*Tamiasciurus hudsonicus*) [61, 70]. Therefore, although our observation of a decline in genetic diversity due to culling could have been expected, it has not, to our knowledge, been demonstrated before in non-native invasive species. One study analysing temporal variation of genetic diversity in an intensively trapped invasive, non-native stoat (*Mustela erminea*) population in New Zealand, found that the genetic diversity remained relatively stable after six years of removal [58]. Given that the signs of genetic instability were observed over a relatively short period (3–4 mink generations), we can conclude that the mink culling affected the populations on KI in the way that was intended.

The causal role of genetic diversity in population extinction or decline is not well documented and is still controversial (see for example [71]), but recently the relationship between microsatellite marker heterozygosity, genome-wide heterozygosity and fitness has been demonstrated [72–74]. The reduction of allelic diversity in a small population may potentially affect population persistence due to, for example, inbreeding depression. Inbreeding depression negatively affects many traits such as survival, or the fecundity of various mammalian populations of endangered species including tigers (*Panthera tigris*), Florida panthers (*Puma concolor*), chimpanzees (*Pan troglodytes*) or harbour seals (*Phoca vitulina*), [75–78]). Similar effects as a result of a decline in genetic diversity were observed in other groups of animals such as amphibians or insects (e.g. [79, 80]). Therefore, if the culling of the mink population on KI decreased genetic variation and heterozygosity, we can predict that the lower genetic variation may have reduced persistence potential in the target area due to inbreeding depression. The relatively small effective population size combined with a relatively high culling pressure makes the mink population on KI more vulnerable to decrease in population size, which confirms the positive effect of mink management. However, we have no direct information about changes in demographic parameters of the mink population on KI to confirm this conjecture.

An understanding of the population genetic structure in management areas is necessary to predict the potential effect of culling on genetic subdivision, and also to inform how culling should be distributed among management units [2]. We observed a strong subdivision between island and mainland populations, as was expected due to the open sea barrier. First, a majority of pairwise comparisons had *F*_ST_ values significantly greater than zero. Second, assignment tests placed more than 85% of individuals into their sampled population. Third, Bayesian clustering methods implemented in STRUCTURE, with no *a priori* information on an individual’s origin, grouped samples according to their sites of origin. Similar results were obtained by DAPC analysis, which also revealed the presence of five clusters in the study sites. Interestingly, the removal of mink increased differentiation between the KI and NC sites. The pairwise *F*_ST_ values were lowest when we compared mink individuals sampled on KI in 2006 to mink sampled at NC in the next years. The *F*_ST_ values increased and were highest when we compared mink from KI sampled in 2011 to mink sampled at NC in all other years. The observed increase in genetic structuring was probably affected by the reduction of the number of migrants from both sites. Furthermore, we also found weak genetic structuring within KI, in different groups of individuals in the eastern and western part of the island archipelago. A total of five clusters of American mink were identified (based on two different analyses) which partially reflected their distribution across the sites, but this also suggested that there were other factors shaping mink genetic structure (most probably history of island colonisation) as individuals from KI were grouped into three clusters in a west-east gradient.

The reduced migration rate from the NC site to KI due to the culling performed on the coast caused the decline of genetic diversity and the increased structuring of mink between these sites. The number of migrants in consecutive years declined (based on Structure assignment). Presumably several factors influence a mink’s ability to traverse the sound and invade the KI. The migrants from the coast were mainly collected on the southern part of the KI archipelago, which is concordant with the prevailing direction of the sea current (from south to north). This suggests that sea current may affect the dispersal potential of mink; individuals from sites on the coast located in the south may have colonized the islands by swimming with the current. This finding also suggests that due to the prevailing surface current direction, from south to north, to further reduce mink colonization of the islands it is advisable to intensify mink culling on the coast located south of KI. The observed pattern exemplifies the usefulness of genetic monitoring to assist in the management of invasive species, by suggesting how to reduce the migration of such species to areas important for biodiversity protection.

Interestingly, the recent migration rate was slightly higher from KI to NC than in the opposite direction. This is a rather unexpected result considering that the coast population of mink is probably larger than the population on KI. One possible explanation could be that the food base is limited on the islands after the bird breeding season. A second explanation could be that individuals (especially males) disperse from the low density islands to find mating opportunities on the mainland. The majority of migrants recorded in this study were males, which confirms this hypothesis. Moreover, male-biased migration was also observed in another American mink population [23], and is characteristic of many mammalian species [81–83]. Male- biased migration might be explained by body size dimorphism in mustelids—larger males can probably swim faster and for a longer distance. Therefore, they are more effective at reaching the islands. However, stoat females are not necessarily inferior to males in swimming speed or endurance [84].

As expected effective population size was smaller on KI than at both coast sites. The upper 95% confidence interval was between two to six times lower on KI than at the coastal sites. Estimating the effective population size with any precision in large populations (>1000 individuals), and using a small number of samples or small number of loci is very challenging [54]. However, for very small populations, even a small number of samples can provide useful and relatively precise estimates [54]. In our study, the small sample size probably affected the estimation of the effective population size. However, increasing the number of loci (in our study to 20) improves the precision of estimation to a greater extent than increasing the sample size [85].

Various methods of estimating the effective population size may provide different results due to different assumptions [54, 85]. In some studies however, using different methods produced very similar values for effective population size; LD is one of the methods where estimates of the effective population size are comparable to other, more direct methods [86]. In our study LD methods gave a two times higher estimation of effective population size than the MC method. Taking these numbers with caution, we may assume that removing mink from the KI population at a level that is similar to the effective population size (about 30 mink per season) may be sufficient to cause substantial decline in genetic diversity, but may not be sufficient to affect effective population size. At the NC site, the number of mink removed was much lower (on average 7 mink per season) than the estimated effective population size, and was possibly not sufficient to affect genetic diversity. Furthermore, immigration of mink from inland may affect the population on the coast. This may suggest that the removal of individuals from the population at a similar level to the effective population size, may be sufficient to reduce the population’s genetic diversity. From a management perspective, estimation of the minimal number of individuals that should be removed to have an effect on a population is a crucial question; therefore, further analysis should be carried out to find the ratio, of number of removed individuals to effective population size, which affects genetic diversity.

## Conclusions

As the negative impact of non-native invasive species (including American mink) on native biodiversity becomes apparent, there is a growing realization of the need for active management to minimize this impact. Control programmes are increasingly used worldwide, and successful eradication has been achieved mainly on islands (e.g. [15]), but also on large areas of the mainland (e.g. [87]). Management of non-native species is usually not done on just one occasion: to be effective, management should be carried out over many years. In the medium term, monitoring of the effects of such actions on the population of invasive species should be carried using genetic approaches as proposed in this paper. Information obtained by analysing culling data, DNA markers, and carcass inspections will become extremely useful management tools to achieve the required long-term local control of such species. In many studies, pre-eradication samples were suggested to be collected and analysed, to evaluate the population structuring and to define management units, and to assess the effects of potential barriers reducing gene flow between groups of sub-populations. Our results illustrate the advantages of a time-series approach applied to a relatively long-lived invasive species. A higher level of temporal variation was observed in the isolated small population, which is probably more affected by genetic drift shaping its genetic structure. Further recognition of the factors that limit or decrease genetic diversity within populations will improve our understanding of the adaptive potential of non-native invasive species. The analysis of temporal samples, the number of mink eradicated per year, and population demographic parameters, may provide a better understanding of the potential effects of culling on an invasive species population and provide the opportunity to evaluate the effectiveness of management.

## Supporting Information

S1 Dataset Microsatellite genotypes of the American mink samples. (XLSX)

S1 Fig Optimization α-score graph. (PDF)

S2 Fig Results of population genetic clustering based on the Bayesian modelling methods in STRUCTURE. The posterior probabilities averaged across 10 replicate runs at each level of K, proposed clusters (left), and the model value of the second order rate of change of the likelihood function (right). Analysis conducted for: all samples (level 1, upper panel), Koster Islands (level 2 –Cluster1, middle panel) and the coast (level 2- Cluster 2, lower panel).
(PDF)

S3 Fig Average proportion of mink assigned to the west and east sites of Koster Islands based on STRUCTURE. Background map: Europe Base Map—Level 1 Provinces, AND Products B.V. and AND Data Ireland Limited, ESRI.
(PDF)

S4 Fig Population genetic clustering results based on the Bayesian Information Criterion (BIC) in relation to the number of clusters identified by the find.cluster function in DAPC analysis. (PDF)

S1 Table Pairwise *F*_ST_ (below diagonal) and harmonic mean estimate D_est_ across loci (above diagonal) comparison between samples taken from three sites (Koster Islands, North and South Coast) in Sweden in 2006–2011. Statistical significance for pairwise *F*_ST_ is given using the adjusted nominal level for multiple comparisons after Bonferroni correction. Sample sizes are given in parentheses. North Coast samples from 2008 and 2009 as well as from 2010 and 2011 were combined.
(PDF)

S2 Table The average proportion of membership for the clusters identified by STRUCTURE and DAPC compared with the sample site of American mink in Sweden N—number of mink analysed. Average membership higher than 0.4 is shown in grey cells.
(PDF)
