# Supplementary figures and images for: Reduced Genetic Diversity and Increased Structure in American Mink on the Swedish Coast following Invasive Species Control
Source: PLoS One. 2016 Jun 22;11(6):e0157972. doi: 10.1371/journal.pone.0157972 (PMC4917106; doi:10.1371/journal.pone.0157972)

## a-score optimisation - spline interpolation

Optimal number of PCs: 7

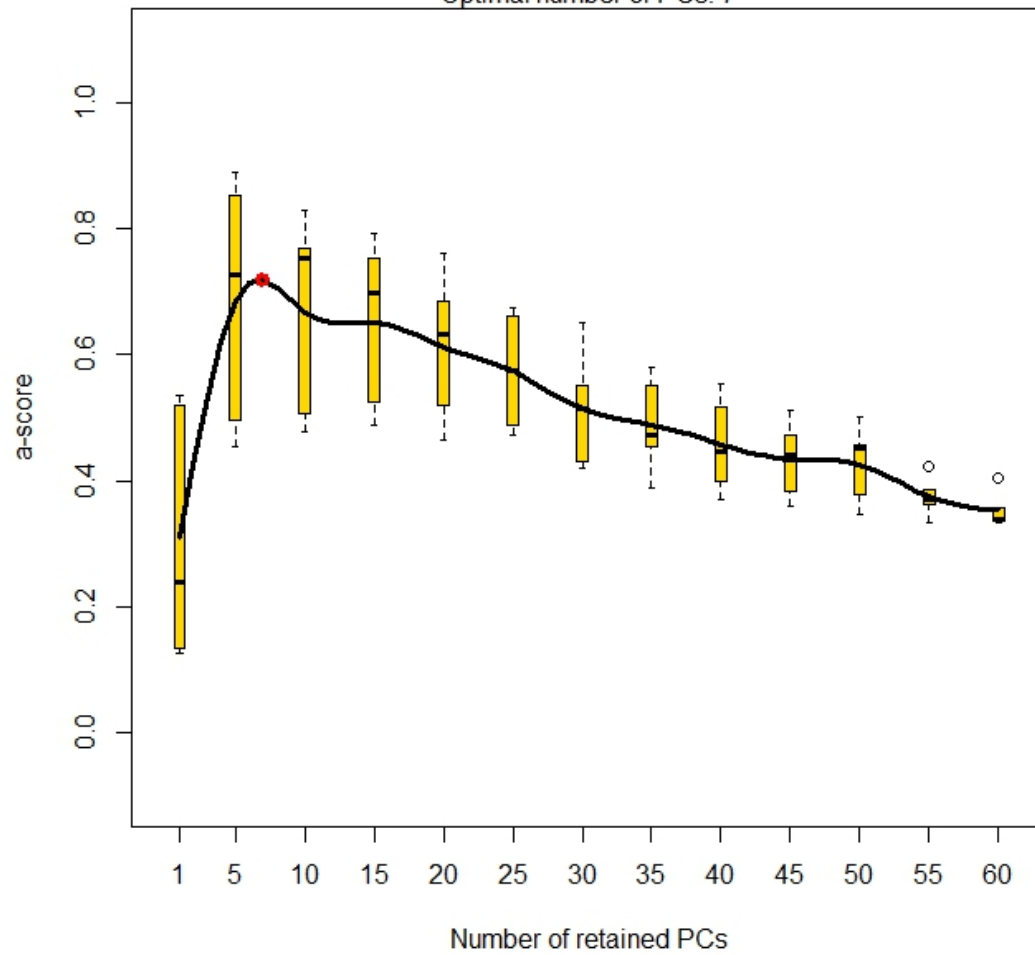

Supplement: S1 Fig — (PDF) [file pone.0157972.s002.pdf]

## Level 1

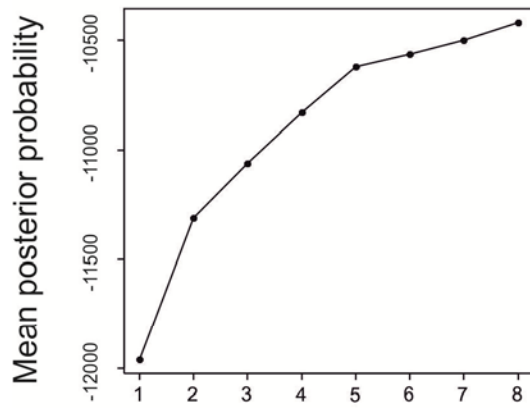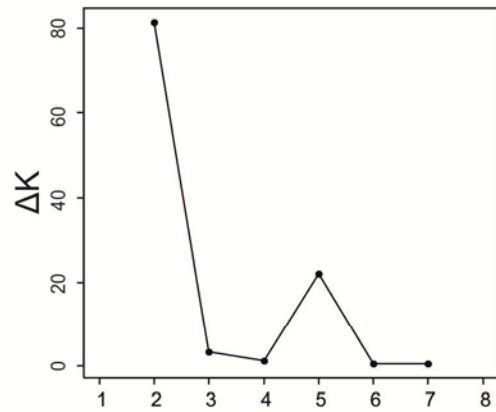

## Level 2- Cluster 1

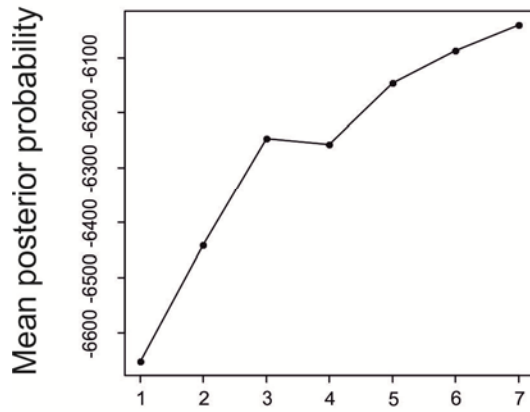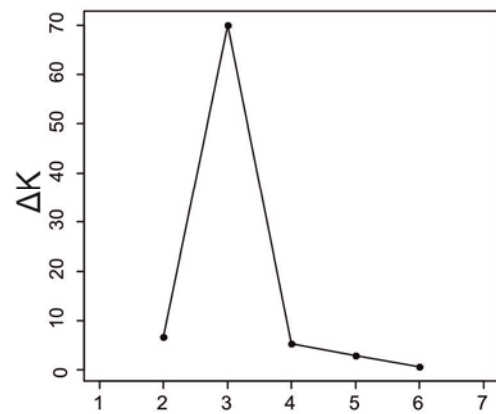

## Level 2- Cluster 2

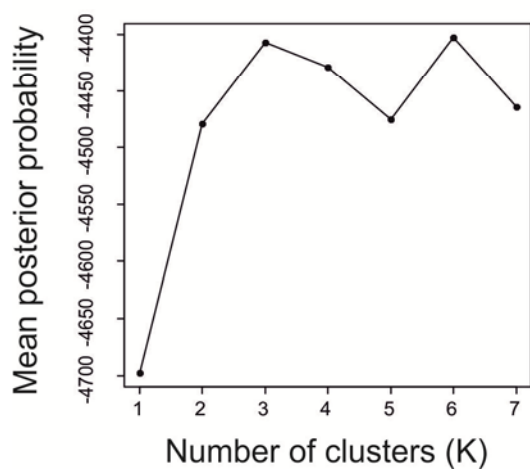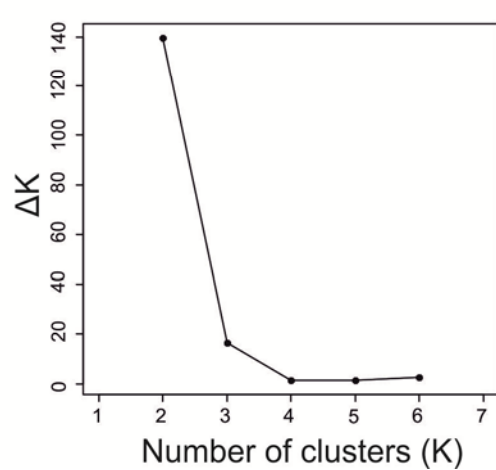

Supplement: S2 Fig — The posterior probabilities averaged across 10 replicate runs at each level of K, proposed clusters (left), and the model value of the second order rate of change of the likelihood function (right). Analysis conducted for: all samples (level 1, upper panel), Koster Islands (level 2 –Cluster1, middle panel) and the coast (level 2- Cluster 2, lower panel). (PDF) [file pone.0157972.s003.pdf]

# Koster Islands

West

East

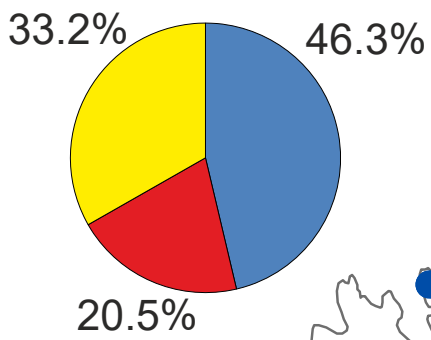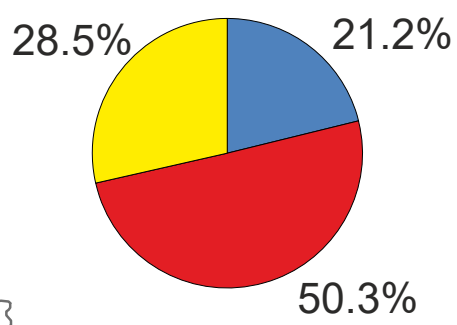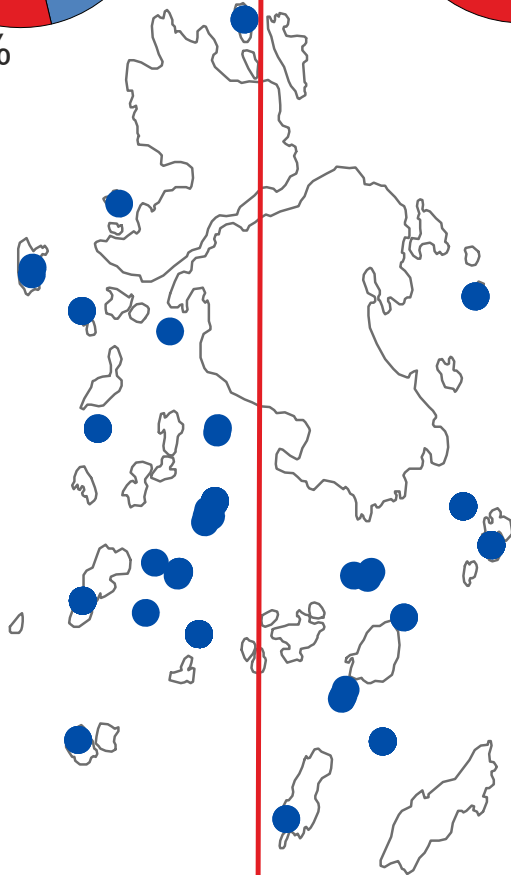

Supplement: S3 Fig — Background map: Europe Base Map—Level 1 Provinces, AND Products B.V. and AND Data Ireland Limited, ESRI. (PDF) [file pone.0157972.s004.pdf]

**Value of BIC  
versus number of clusters**

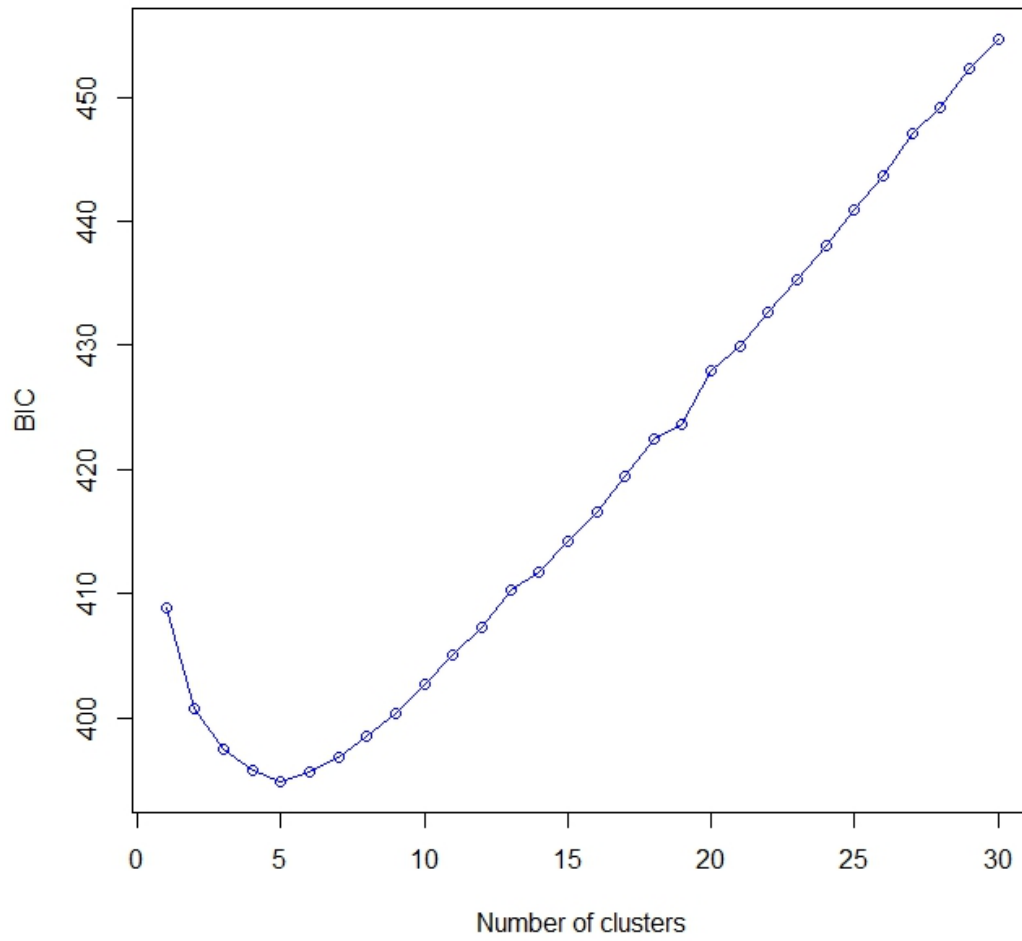

Supplement: S4 Fig — (PDF) [file pone.0157972.s005.pdf]
