# Supplementary material for: Reduced Genetic Diversity and Increased Structure in American Mink on the Swedish Coast following Invasive Species Control
Source: PLoS One. 2016 Jun 22;11(6):e0157972. doi: 10.1371/journal.pone.0157972 (PMC4917106; doi:10.1371/journal.pone.0157972)
Supplement: S1 Table — Statistical significance for pairwise FST is given using the adjusted nominal level for multiple comparisons after Bonferroni correction. Sample sizes are given in parentheses. North Coast samples from 2008 and 2009 as well as from 2010 and 2011 were combined. (PDF) [file pone.0157972.s006.pdf]

S1 Table. Pairwise F_{ST} (below diagonal) and harmonic mean estimate D_{est} across loci (above diagonal) comparison between samples taken from three sites (Koster Islands, North and South Coast) in Sweden in 2006-2011. Statistical significance for pairwise F_{ST} is given using the adjusted nominal level for multiple comparisons after Bonferroni correction. Sample sizes are given in parentheses. North Coast samples from 2008 and 2009 as well as from 2010 and 2011 were combined.

Site and year	Koster Islands						North Coast				South Coast
	KI 2006 (30)	KI 2007 (24)	KI 2008 (29)	KI 2009 (11)	KI 2010 (10)	KI 2011 (28)	NC 2006 (10)	NC 2007 (15)	NC 2008 (12)	NC 2010 (9)	SC 2010 (27)
KI 2006	--	0.0014	0.0289	0.0099	0.0247	0.0422	0.0387	0.0572	0.0935	0.0230	0.1343
KI 2007	0.0037	--	0.0001	-0.0002	0.0003	0.0105	0.0524	0.0702	0.0933	0.0503	0.1561
KI 2008	0.0259**	0.0025	--	-0.0008	0.0001	0.0053	0.0573	0.0596	0.0815	0.0382	0.1676
KI 2009	0.0195	-0.0022	-0.0036	--	0.0005	0.0000	0.0525	0.0558	0.0753	0.0292	0.1576
KI 2010	0.0220	0.0066	0.0056	0.0082	--	0.0000	0.0764	0.0773	0.1027	0.0441	0.1505
KI 2011	0.0349***	0.0174**	0.0125**	0.0002	0.0032	--	0.1120	0.0881	0.1071	0.0726	0.1736
NC 2006	0.0313	0.0420**	0.0578	0.0584*	0.0735*	0.0909**	--	0.0011	0.0146	-0.0001	0.0979
NC 2007	0.0418**	0.0556***	0.0628***	0.0593***	0.0741***	0.0816***	0.0063	--	0.0028	0.0052	0.1160
NC 2008	0.0652**	0.0713***	0.0737***	0.0712**	0.0789*	0.0903***	0.0262	0.0103	--	0.0046	0.1235
NC 2010	0.0291	0.0376**	0.0417*	0.0509**	0.0537*	0.0722***	-0.0070	0.0192	0.0212	--	0.1178
SC 2010	0.0771***	0.0969***	0.1111***	0.1019***	0.1074***	0.1160***	0.0651**	0.0758***	0.0917***	0.0727***	--
