# Supplementary material for: Reduced Genetic Diversity and Increased Structure in American Mink on the Swedish Coast following Invasive Species Control
Source: PLoS One. 2016 Jun 22;11(6):e0157972. doi: 10.1371/journal.pone.0157972 (PMC4917106; doi:10.1371/journal.pone.0157972)
Supplement: S2 Table — Average membership higher than 0.4 is shown in grey cells. (PDF) [file pone.0157972.s007.pdf]

S2 Table. The average proportion of membership for the clusters identified by STRUCTURE and DAPC compared with the sample site of American mink in Sweden N - number of mink analysed. Average membership higher than 0.4 is shown in grey cells.

Site	Code and Year	N	Structure - Level 1		Structure - Level 2			DAPC				
			**Cluster 1**	**Cluster 2**	**Cluster 1a**	**Cluster 1b**	**Cluster 1c**	**Cluster 1**	**Cluster 2**	**Cluster 3**	**Cluster 4**	**Cluster 5**
Koster Islands	KI 2006	30	0.719	0.281	0.612	0.210	0.178	0.591	0.169	0.153	0.070	0.017
	KI 2007	24	0.838	0.162	0.354	0.296	0.350	0.335	0.332	0.288	0.040	0.005
	KI 2008	29	0.853	0.147	0.171	0.448	0.381	0.277	0.497	0.182	0.044	0.000
	KI 2009	11	0.904	0.096	0.179	0.396	0.425	0.383	0.404	0.204	0.008	0.001
	KI 2010	10	0.943	0.058	0.266	0.480	0.254	0.245	0.512	0.243	0.000	0.000
	KI 2011	28	0.920	0.080	0.247	0.436	0.317	0.299	0.557	0.123	0.017	0.005
					**Cluster 2a**	**Cluster 2b**						
North Coast	NC 2006	10	0.188	0.812	0.964	0.036		0.084	0.017	0.253	0.640	0.005
	NC 2007	15	0.197	0.803	0.903	0.097		0.086	0.003	0.092	0.765	0.054
	NC 2008-2009	12	0.181	0.819	0.917	0.083		0.085	0.084	0.014	0.814	0.002
	NC 2010-2011	9	0.234	0.766	0.977	0.023		0.117	0.114	0.208	0.551	0.009
South Coast	SC 2010-2011	27	0.048	0.952	0.071	0.929		0.000	0.000	0.006	0.042	0.951
